# Article antidiabetic potential of peanut oil: inhibiting *α*-amylase and *α*-glucosidase using identified phytochemicals through *in vitro* and *in silico* studies

**DOI:** 10.3389/fnut.2025.1592468

**Published:** 2025-10-07

**Authors:** Djamila Benouchenne, Hanène Djeghim, Ouided Benslama, Huda Alsaeedi, David Cornu, Mikhael Bechelany, Ahmed Barhoum

**Affiliations:** ^1^Laboratoire de Génétique, Biochimie et Biotechnologie végétale, Faculté des Sciences de la Nature et de la vie, Université des Frères Mentouri Constantine 1, Constantine, Algeria; ^2^Higher National School of Biotechnology Taoufik Khaznadar, Nouveau Pôle Universitaire Ali Mendjli, Constantine, Algeria; ^3^Biochemistry Laboratory, Biotechnology Research Center (CRBt), Constantine, Algeria; ^4^Department of Natural and Life Sciences, Faculty of Exact Sciences and Natural and Life Sciences, Larbi Ben M’Hidi University, Oum El Bouaghi, Algeria; ^5^Department of Chemistry, College of Science, King Saud University, Riyadh, Saudi Arabia; ^6^Institut Européen des Membranes, University Montpellier, Montpellier, France; ^7^NanoStruc Research Group, Chemistry Department, Faculty of Science, Helwan University, Cairo, Egypt; ^8^School of Chemical and BioPharmaceutical Sciences, Technological University Dublin, Dublin, Ireland

**Keywords:** α-amylase inhibition, α-glucosidase inhibition, peanut oil bioactivity, antidiabetic therapy, molecular docking simulation, *in vitro* analysis

## Abstract

**Background:**

Peanut oil is recognized for its mild flavor, high phytochemical content, medicinal potential, and other health advantages.

**Objective:**

This study, for the first time, evaluates the antidiabetic potential of peanut oil, known for its high phytochemical content and medicinal properties.

**Methods:**

The oil, collected from the El Oued region of Algeria, was extracted using the Soxhlet technique with n-hexane as the solvent. The obtained oil was subjected to gas chromatography–mass spectrometry (GC/MS) analysis. The antidiabetic effect *in vitro* was examined by inhibiting *α*-amylase and α-glucosidase enzymes. The molecular docking was performed using Molecular Operating Environment (MOE) software to assess the inhibitory potential of 20 identified phytochemical compounds against *α*-amylase (PDB ID: 2QV4) and α-glucosidase (PDB ID: 5NN8).

**Results:**

The oil is showing an inhibitory activity against α-amylase and α-glucosidase. Twenty fatty acid compounds representing 99.9% of the oil content were classified by gas chromatography–mass spectrometry (GC/MS) analysis into saturated fatty acids (SFA), monounsaturated fatty acids (MUFA), and polyunsaturated fatty acids (PUFA). Peanut oil demonstrated significant *α*-amylase inhibitory activity with an IC_50_ value of 228.23 ± 5.68 μg/mL, surpassing the standard inhibitor, acarbose, which had an IC_50_ of 3650.93 ± 10.70 μg/mL. Conversely, the α-glucosidase inhibition by peanut oil was less pronounced, with an IC_50_ value exceeding 1,000 μg/mL. Acarbose exhibited a much stronger effect with an IC_50_ of 405.77 ± 34.83 μg/mL. The molecular docking outcomes stated that stearic acid had a binding energy of −7.5729 kcal/mol and formed hydrogen bonds with residues like Gly164, Asn105, and Ala106, along with hydrophobic interactions with His201, Leu162, Tyr62, Leu165, and Trp59 in *α*-amylase inhibitory while in α-glusosidase inhibitory apt, the data revealed that compounds such as oxiraneoctanoic acid, 3-octyl, exhibited a favorable binding energy of −6.5120 kcal/mol and formed hydrogen bonds with key residues His674 and Asp616.

**Conclusion:**

These findings suggest that while peanut oil holds promise as a natural *α*-amylase inhibitor, its effect on *α*-glucosidase is relatively modest compared to the synthetic standard. Further research is recommended to explore the potential synergistic effects of peanut oil’s components for enhanced enzyme inhibition.

## 1 Introduction

Diabetes mellitus is associated with chronic hyperglycemia. It is still one of the biggest global health threats. According to Sun et al. (1), as of 2021, 537 million adults aged 20–79 years have diabetes, and that number is estimated to rise to 783 million in 2045 (1). There are two major classifications of diabetes: Type 1 diabetes is caused by the autoimmune destruction of pancreatic beta cells that leads to a deficiency of insulin, and Type 2, which is characterized by relative insulin deficit and insulin resistance. Poorly controlled diabetes results in several debilitating adverse effects, which include cardiovascular diseases, diabetic nephropathy, retinopathy, and neuropathy (2). Standard management steps such as lifestyle changes, oral hypoglycemic agents, and insulin treatment, were undertaken (3). However, even with these options, attaining and sustaining glycemic control is difficult for many individuals, thereby underscoring the necessity for alternative and adjunctive treatment methods with minimal adverse effects (4).

Medicinal plants have long been recognized for their role in managing diabetes, primarily due to their diverse bioactive compounds that regulate glucose metabolism and enhance insulin sensitivity (38, 40). Traditional systems such as Ayurveda and Traditional Chinese Medicine have utilized herbs like cinnamon, bitter melon, and fenugreek for their antidiabetic effects (5, 37). Beyond these commonly known plants, recent studies have highlighted the potential of peanut (*Arachis hypogaea* L.) and its derivatives. Peanut oil, in particular, is valued not only for its mild flavor but also for its high content of phytochemicals with medicinal properties (6). Enzymatically hydrolyzed peanut proteins have demonstrated the ability to inhibit *α*-amylase and α-glucosidase, two key enzymes involved in carbohydrate digestion (7). Additionally, peanut extracts have been shown to significantly lower fasting blood glucose and HbA1c levels in diabetic animal models, likely due to their monounsaturated fatty acids and antioxidant properties (8). Similarly, peanut shell polyphenol extracts (PSPE) have exhibited hypoglycemic effects comparable to metformin in high-fat diet/streptozotocin-induced diabetes models, while maintaining low toxicity (7).

These findings align with broader investigations into enzyme inhibition as a mechanism of antidiabetic action in various medicinal plants. Compounds that inhibit *α*- and *β*-amylase and α-glucosidase can delay glucose absorption, thus controlling postprandial blood glucose levels (9). In this context, *in silico* molecular docking studies have played a crucial role by simulating interactions between plant-derived compounds, especially flavonoids and polyphenols and digestive enzymes, often revealing strong binding affinities and potential inhibitory activity (10, 11). These computational insights are further validated by *in vitro* assays. For example, extracts of *Gymnema sylvestre* showed significant inhibition of both *α*-amylase and α-glucosidase, with IC50 values of 45 μg/mL and 38 μg/mL, respectively (12). Together, these studies support the promising role of plant-based interventions, including peanut oil and its derivatives, in the management of type 2 diabetes through enzyme inhibition and oxidative stress modulation.

To the best of our knowledge, this is the first study to explore the antidiabetic potential of peanut oil extracted from the El Oued region of Algeria, focusing on its inhibitory effects against *α*-amylase and α-glucosidase, i.e., two key enzymes involved in carbohydrate digestion. A combined *in vitro* and *in silico* approach was employed to evaluate the oil’s biochemical activity and molecular interactions. The oil was extracted using Soxhlet extraction with n-hexane and characterized by gas chromatography–mass spectrometry (GC/MS), which identified 20 major fatty acids accounting for 99.9% of the total composition. The *α*-amylase inhibitory assay was carried out on oil samples at varying concentrations to measure reductions in maltose production, thereby assessing the oil’s capacity to inhibit starch digestion. Likewise, the α-glucosidase 4-nitrophenyl-α-D-glucopyranoside assay measured p-nitrophenol release to evaluate inhibition of disaccharide breakdown, offering complementary evidence of the oil’s efficacy in reducing postprandial glucose release. Molecular docking simulations using MOE software (PDB: 2QV4 for *α*-amylase and 5NN8 for α-glucosidase) predicted binding affinities and mapped key hydrogen bond and hydrophobic interactions. These findings provide mechanistic insight into the observed *in vitro* enzyme inhibition and highlight the therapeutic promise of peanut oil as a natural agent for managing type 2 diabetes.

## 2 Materials and methods

### 2.1 Chemicals and reagents

The following chemicals and reagents were used: α-Glucosidase from *Saccharomyces cerevisiae* (≥10 units/mg protein, Sigma-Aldrich), 4-nitrophenyl α-D-glucopyranoside (pNPG, ≥99%, Sigma-Aldrich), α-Amylase from *Aspergillus oryzae* (≥5 units/mg solid, Sigma-Aldrich), acarbose (95%, VWR), potato starch (Thermo Fisher), Na₂HPO₄ (ACS reagent, ≥99.0%, Sigma-Aldrich), NaH₂PO₄ (ReagentPlus®, ≥99.0%, Sigma-Aldrich), NaCl (≥98%, TECHNICAL), iodine (ACS reagent, ≥99.8%, solid, Sigma-Aldrich), potassium iodide (ACS reagent, ≥99.0%, Sigma-Aldrich), methanol (≥99.8%, HPLC grade, Sigma-Aldrich), and hexane (≥97.0%, HPLC grade, Sigma-Aldrich).

### 2.2 Plant materials collection and oil extract

Peanut seeds were obtained from the El Oued region in Algeria, a region known to produce high quality peanuts due to optimal growing conditions (13). The standard herbarium code for the peanut (*Arachis hypogaea* L.) is “ARHHY,” as designated by the European and Mediterranean Plant Protection Organization (EPPO). Samples were kept in paper bags at 4 °C to control fungal growth, as well as metabolic activity, until processing. Peanut oil was extracted from peanut seeds with a Soxhlet apparatus according to (14). One gram of dry seeds was ground into a coarse powder. Then, the powder was subjected to Soxhlet extraction (FOSS Soxtec™ 8,000) using n-hexane (≥98% purity, Sigma-Aldrich) as the solvent. Extraction lasted for 6 h, which is sufficient for complete oil recovery. For each 1 g of peanut kernels placed in the thimbles, 25 mL of n-hexane was added to the extraction vessels. Hexane was selected as the extraction solvent because it is the most widely used for oil recovery from lipid-rich matrices such as peanuts, providing high extraction efficiency, reproducibility, and selective extraction of non-polar compounds. Previous studies have demonstrated that hexane ensures maximum oil yield and efficient recovery in peanuts compared to other solvents (15), and that n-hexane is routinely employed for residual oil extraction in peanut processing after mechanical pressing (16). Moreover, its narrow boiling range and high solubilizing capacity make hexane the preferred solvent in edible oilseed extraction and lipid profiling applications (17). The obtained oil was kept for further analyses. Oil content was determined through measurement of weight differences between an empty extraction cartridge and cartridge following extraction of oil.

### 2.3 GC–MS analysis of peanut oil

Gas Chromatography–Mass Spectrometry (GC–MS) was used in determining the chemical composition of the extracted peanut oil. It has been used in preference to HPLC due to its superior capacity to analyze non-polar compounds like fatty acids and bioactive lipids, major constituents of the oil in question (34, 35, 39). Peanut oil is a very complex mixture of volatile and semi-volatile compounds, including saturated, monounsaturated, and polyunsaturated fatty acids that can be efficiently separated, identified, and quantified by GC–MS according to their mass spectra. In contrast, HPLC is better suited for polar compounds, making GC–MS the more appropriate choice for this analysis. The GC–MS analysis was conducted with an HP 6890 GC system coupled with a 5,973 mass spectrometer (Hewlett Packard). The column specifications were 60 m × 0.32 mm i.d. × 0.25 μm film thickness, with helium as the carrier gas at a flow rate of 1 mL/min. The temperature settings for the column, detector, and injector were 225 °C, 245 °C, and 250 °C, respectively. Compounds were identified by comparing retention times with reference standards from NIST and Wiley mass spectral libraries and matching fragmentation patterns.

### 2.4 *In silico* prediction of anti-hyperglycemic activity

Molecular docking simulations were performed using the Molecular Operating Environment (MOE) software, version 2015.10, to investigate the inhibitory potential of 20 identified phytochemical compounds against two key enzymes involved in glucose metabolism, namely *α*-amylase (PDB ID: 2QV4, human pancreatic enzyme) and α-glucosidase (PDB ID: 5NN8, human lysosomal enzyme). Phytochemical compounds molecular structures were downloaded from PubChem, https://pubchem.ncbi.nlm.nih.gov, while the enzyme structures were retrieved from the Protein Data Bank, PDB, https://www.rcsb.org. In preparation for docking, the enzyme structures were prepared by removing water molecules, adding hydrogen atoms, and ensuring the proper protonation states. Added in were missing residues and atoms, and the enzyme structures were energy-minimized to resolve steric clashes. Energy minimization using the MMFF94 force field was performed for the ligand structures to ensure that the conformations are stable. For each ligand-enzyme complex, 10 docking poses were generated. The validation of the docking protocol was performed by reproducing the co-crystallized ligand-binding pose for each enzyme that gave RMSD values of 0.745 Å for *α*-amylase and 0.620 Å for *α*-glucosidase, respectively, thus confirming that the docking protocol is correct. Post-docking analysis was performed using Discovery Studio software, Version 2024.1.0, with a focus on key interactions such as hydrogen bonding, hydrophobic interactions, and electrostatic interactions. Each ligand’s binding affinity was then analyzed based on binding energy and interaction profiles with critical amino acid residues in both enzyme active sites. The computational resource employed for docking simulations was a computer system consisting of an Intel Xeon processor, 32 GB of RAM, and an NVIDIA Quadro (M2000M) GPU. The above *in silico* analysis gives insight into the possible inhibiting properties of the identified phytochemicals of peanut oil against *α*-amylase and α-glucosidase, which are potential anti-hyperglycemic agents of natural origin.

### 2.5 *In vitro* α-amylase inhibition assay

α-Amylase inhibition assay was performed using the IKI method as reported in the literature (18). The peanut oil at a series of dilutions was subjected to *α*-amylase in 96-well format. Following 10 min pre-incubation at 37 °C, the enzyme assay was started by the addition of a starch substrate. The reaction was stopped with HCl and further color development was performed with iodine/potassium iodide. Absorbance was measured at 630 nm on a PerkinElmer EnSpire microplate reader. Acarbose was the positive control. The inhibition of *α*-amylase can be calculated using the following equation:


$$\text{Inhibition}\%=1-[\frac{\left(\mathrm{Ac}-\mathrm{Ae})-(\mathrm{As}-\mathrm{Ab}\right)}{\left(\mathrm{Ac}-\mathrm{Ae}\right)}]$$

$$\begin{array}{l} \mathrm{Ac}=\text{Absorbance}\;[\text{Starch}+\mathrm{IKI}+\mathrm{HCl}+\text{Methanol}\\ +\text{Volume of Enzyme buffer}] \end{array}$$

Ae=Absorbance[Enzyme+Starch+IKI+HCl+Methanol]

As=Absorbance[Enzyme+Peanut Oil+Starch+IKI+HCl]

Ab=Absorbance[Peanut Oil+IKI+125μLof buffer]

### 2.6 *In vitro* α-glucosidase inhibition assay

The α-glucosidase inhibition assay was performed based on the method described by Lordan et al. (19) with minor modifications. Peanut oil at different concentrations was incubated with α-glucosidase and pNPG substrate in a 96-well microplate at 37 °C. After 30 min, absorbance was measured at 405 nm using a microplate reader. Acarbose was used as a positive control. The % inhibition of α-glucosidase was calculated as:


$$\text{Inhibition}\%=[\frac{\mathrm{Abs}\;\text{of Peanut Oil}-\mathrm{Abs}\;\text{of Blanc}}{\mathrm{Abs}\;\text{of Control}}\;]*100$$

Absof Blanc=Absorbance[Enzyme+Substrate+Methanol]

$$\begin{array}{l} \mathrm{Abs}\;\text{of}\;\text{Control}=\text{Absorbance}\\ \left[\text{Substrate}+\text{Peanut Oil}+\text{enzyme buffer}\right] \end{array}$$

### 2.7 Statistical analysis

Experiments were performed in triplicate. All data generated were analyzed by Tukey’s test for significant difference at mean values. A *p*-value < 0.05 is considered to be significant. For all statistical analysis, XLSTAT software (version 2016) has been used.

## 3 Results

### 3.1 Oil yield and composition

**Figure 1:**
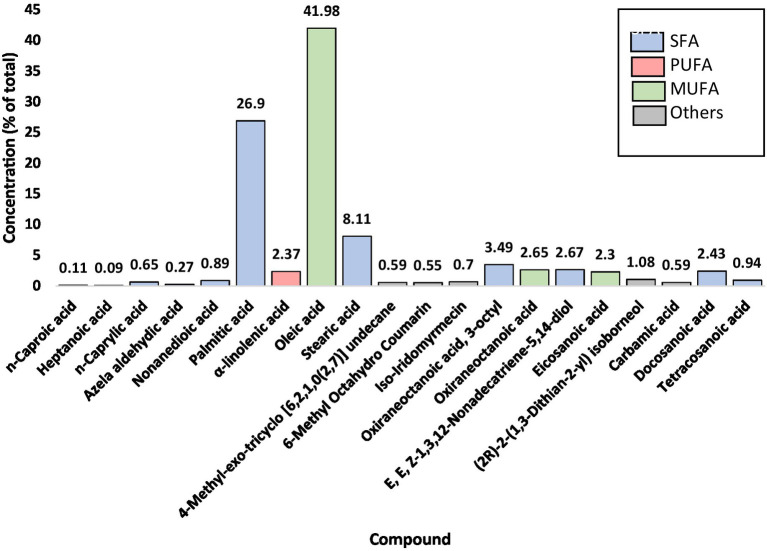
Identified compounds of peanut oil (*Arachis hypogaea* L.).

**Figure 2:**
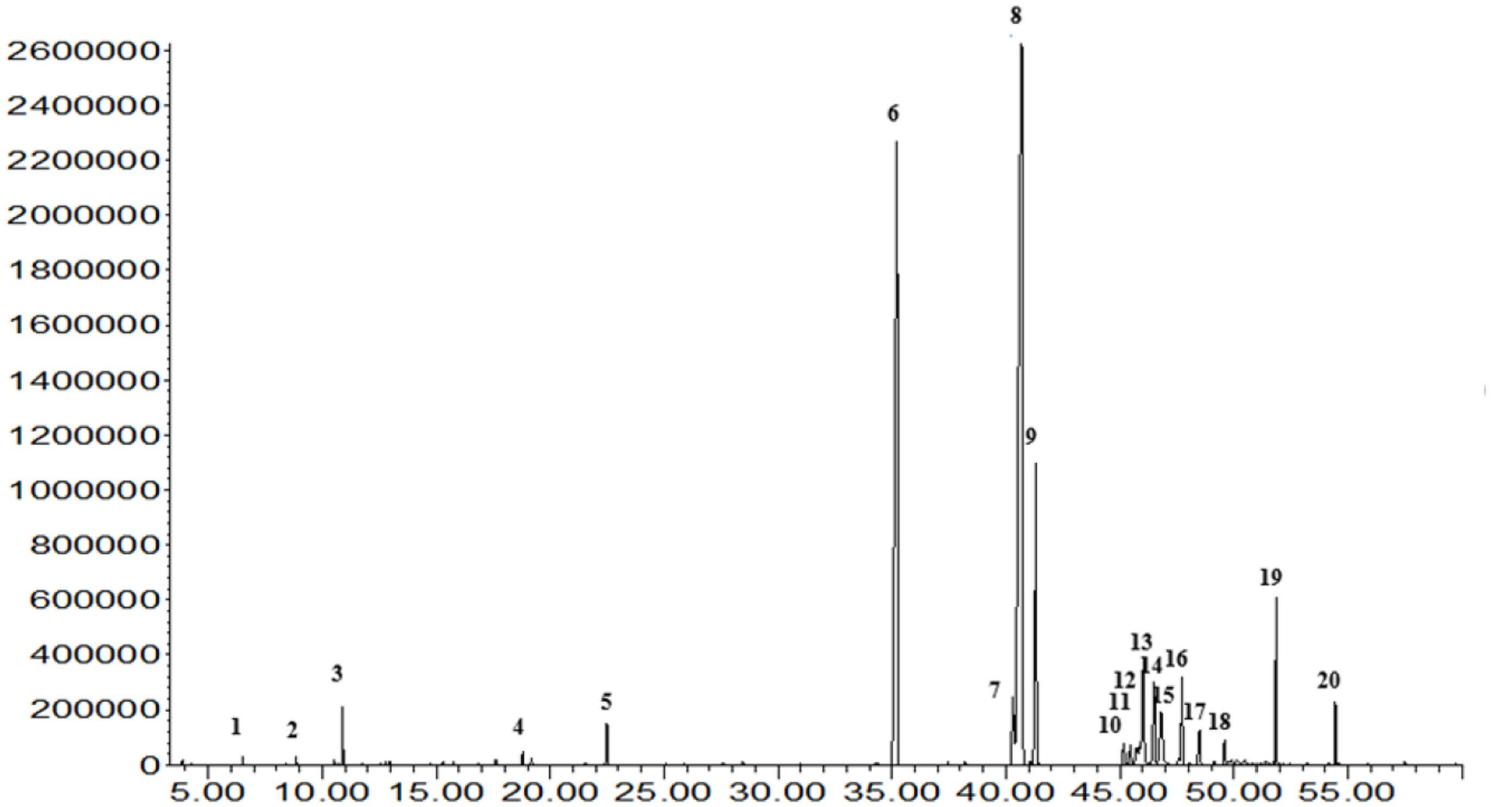
The GC–MS analysis of oil extracted from Algerian peanut (*Arachis hypogaea* L.) cultivated in the El Oued region provides a detailed chemical profile, identifying its various bioactive compounds.

The extraction of peanut oil from the El Oued region resulted in a significant yield of 51.85%, underscoring the rich oil content of the locally sourced peanuts. GC–MS analysis identified 20 fatty acid compounds, collectively accounting for 99.9% of the oil’s composition (Figure 1, Figure 2; Supplementary Figure S2). These compounds were divided into three groups: SFA, MUFA, and PUFA. The total amount of SFA was 47.18%; among these, palmitic acid and stearic acid predominated, representing 26.90 and 8.11%, respectively. MUFAs made up 46.94% of the oil; among them, oleic acid at 41.98% [Supplementary Figure S2 (8)] was the major compound, potentially contributing to the cardiovascular benefits of the oil. The polyunsaturated fatty acids represented a smaller portion, at 2.38%, with *α*-linolenic acid [Supplementary Figure S2 (7)] as a notable component due to its recognized anti-inflammatory properties. This balanced distribution of SFAs, MUFAs, and PUFAs not only imparts stability on peanut oil but also its putative health-promoting properties. Besides, these minor compounds in the oil-like oxiraneoctanoic acid (3.50%) Supplementary Figure S2 (14), and docosanoic acid (2.43%) Supplementary Figure S2 (19) have the potential to explain the bioactive properties of this oil, specifically for enzyme inhibition activity. The GC–MS chemical profile of the extracted oil from the El Oued region, including the calculated Kovats indices and identification details of the detected compounds, is presented in Supplementary Table S1. As well as the mass spectrum of the compounds determined in the oil are illustrated by Supplementary Figure S2.

### 3.2 *In silico* prediction of anti-hyperglycemic activity

In this study, 20 phytochemical compounds from peanut oil were subjected to docking analysis against *α*-amylase and α-glucosidase enzymes. The results provided important insights into the anti-hyperglycemic activity of the tested compounds.

#### 3.2.1 α-amylase inhibitory ability

**Figure 3:**
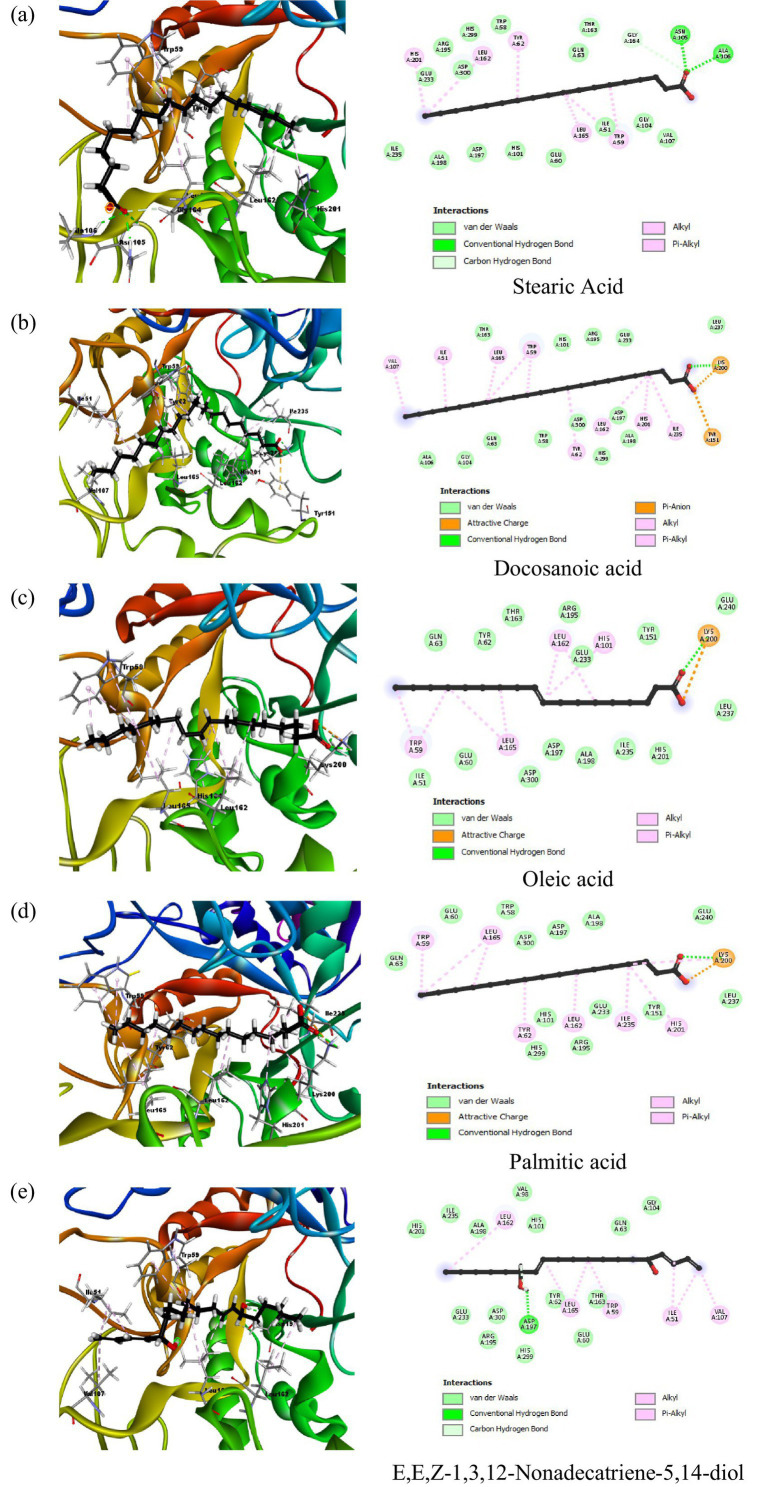
2D and 3D interaction profiles of the top five active compounds from peanut oil with α-amylase (PDB ID: 2QV4), showing key binding interactions at the enzyme’s active site. (**a**) Stearic acid, (**b**) Docosanoic acid, (**c**) Oleic acid, (**d**) Palmitic acid, (**e**) E,E,Z-1,3,12-Nonadecatriene-5,14-diol.

Docking results for α-amylase inhibition (PDB ID: 2QV4) showed that the co-crystallized ligand (QV4) had the lowest binding energy of −10.2266 kcal/mol, indicating a strong inhibitory interaction with the enzyme. This was supported by multiple hydrogen bonds with residues like Ala106, Gly104, and Asp300, and strong electrostatic interactions with residues such as Glu233, Asp197, and Asp300. These interactions suggest robust binding, which is crucial for the effective inhibition of α-amylase. Acarbose standard displayed a similarly strong binding energy of −8.6659 kcal/mol, forming hydrogen bonds with key residues like Glu233 and Asp300, further confirming its inhibitory potential. Among the phytochemicals tested, stearic acid exhibited the highest inhibitory potential with a binding energy of −7.5729 kcal/mol. This was through hydrogen bonding with Gly164 and Asn105, with His201 and Leu162 involved in hydrophobic interactions (Figure 3). The rest of the compounds, representing docosanoic acid, oleic acid, and palmitic acid, have showed binding energies between −7.1463 and −6.4778 kcal/mol, reflecting medium inhibitory potency due to hydrogen bonding with Lys200 and also hydrophobic and electrostatic interactions. All of these compounds showed considerable binding; however, not as strong inhibitions compared to acarbose probably due to weaker electrostatic interactions with important residues. Detailed docking results of compounds in peanut oil against alpha-amylase (2QV4) are shown in Supplementary Table S2.

In addition to these fatty acids, *α*-linolenic acid, eicosanoic acid, tetracosanoic acid, and nonanedioic acid also displayed interactions with the catalytic pocket of α-amylase. In particular, α-linolenic acid showed multiple hydrophobic contacts with Leu165, His305, His101, Leu162, Trp59, and Trp58, along with an electrostatic interaction with Lys200. Eicosanoic and tetracosanoic acids also interacted with Lys200, while nonanedioic acid formed electrostatic contacts with Lys200 and His101. These findings indicate that, beyond acarbose, several fatty acids from peanut oil share common binding modes with key residues such as Lys200, Asp197, and His residues including His101, His201, His299, and His305 within the active site, thereby supporting their role in *α*-amylase inhibition.

#### 3.2.2 α-glucosidase inhibition potential

**Figure 4:**
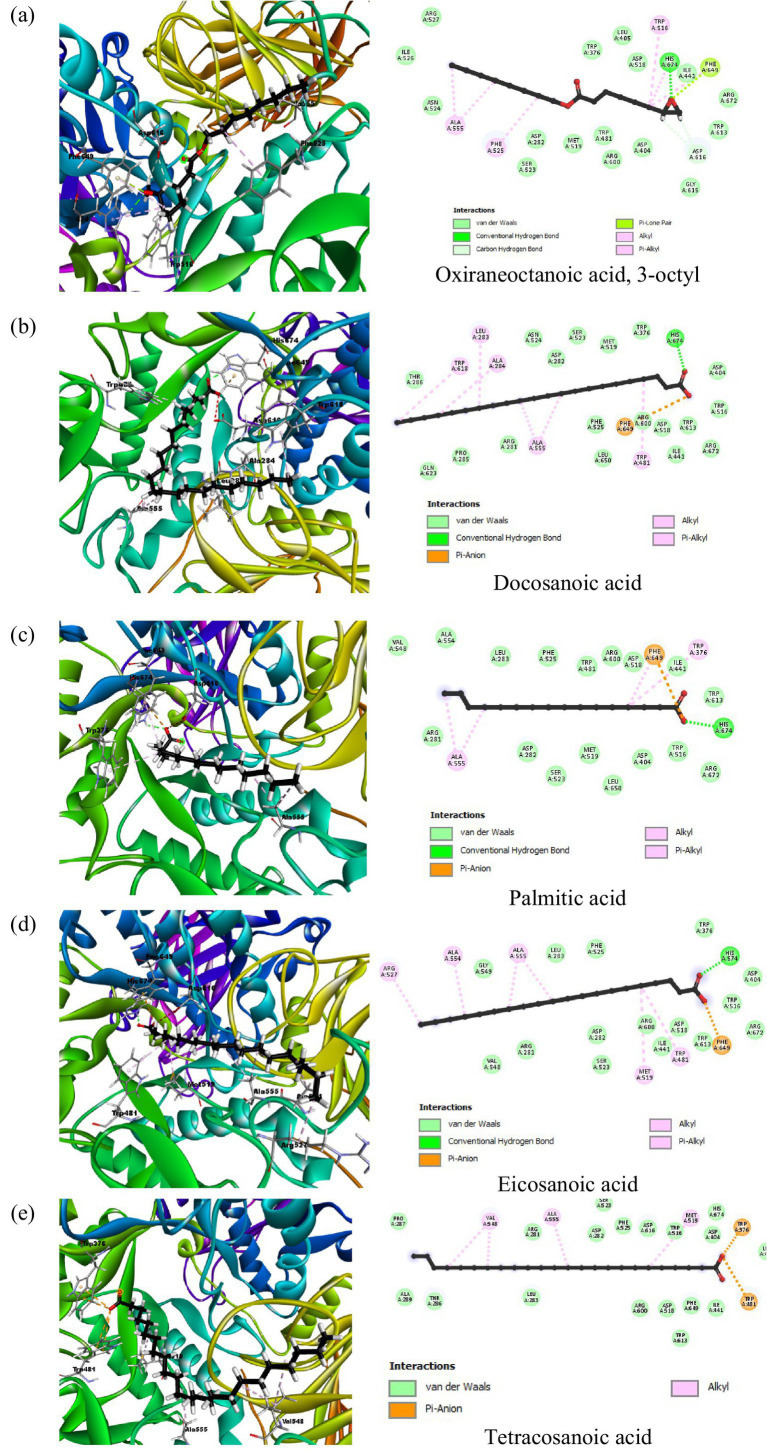
2D and 3D interaction profiles of the top five active compounds from peanut oil with the active sites of α-glucosidase (PDB ID: 5NN8), showing key binding interactions at the enzyme’s active site. (**a**) Oxiraneoctanoic acid, 3-octyl, (**b**) Docosanoic acid, (**c**) Palmitic acid, (**d**) Eicosanoic acid, (**e**) Tetracosanoic acid.

The docking analysis for α-glucosidase, PDB ID: 5NN8, had shown the binding energy of its co-crystallized ligand acarbose to be −7.8732 kcal/mol. Acarbose showed extensive hydrogen bonding interactions with important catalytic residues such as Asp616, Asp518, and Asp404 implicated in the hydrolytic function of the enzyme. These interactions point toward strong inhibition by acarbose, consistent with its clinical use as an inhibitor of this enzyme, α-glucosidase. Among the phytochemical compounds, oxiraneoctanoic acid, 3-octyl, had the highest binding energy of −6.5120 kcal/mol, interacting through hydrogen bonds with His674 and Asp616, two residues important for the catalytic activity of α-glucosidase. Docosanoic acid and palmitic acid also showed very strong inhibitory potentials with binding energies of −6.3286 and −6.2753 kcal/mol, respectively. These compounds interacted mainly through hydrogen bonding with His674 and also showed hydrophobic interactions with nearby residues like Trp481 and Ala555 (Figure 4). Eicosanoic acid showed a binding energy of −6.2354 kcal/mol and formed hydrogen bonds with His674, in addition to hydrophobic interactions with residues such as Arg527 and Ala555. Its binding was not directly concerned with Asp616, but being situated in the active site, it likely acts as a competitive inhibitor since it hinders the access of substrates to the active site. Tetracosanoic acid had the weakest binding energy of 5.8252 kcal/mol, forming no hydrogen bonds with main catalytic residues; hence, its action as an inhibitor is probably weak. The results obtained from the compounds of peanut oil docked on alpha-glucosidase (5NN8) are presented in Supplementary Table S3.

### 3.3 *In vitro* evaluation of anti-hyperglycemic activity

The aim of this section is to show the results from an *in vitro* evaluation regarding the anti-hyperglycemic activity of El Oued peanut oil, related to its efficiency in inhibiting two key enzymes involved in carbohydrate digestion: namely, *α*-amylase and α-glucosidase. These two enzymes, responsible for degrading carbohydrates to fewer complex sugars, may have their impact on the blood glucose levels. The peanut oil’s inhibiting action against both enzymes was also reviewed for its potentially therapeutic benefits concerning hyperglycemia management.

#### 3.3.1 α-amylase inhibitory ability

On α-amylase, peanut oil showed a significant inhibition percentage, in a dose-dependent relationship. The corresponding oil exhibited very good inhibiting capability, showing an IC_50_ of 228.23 ± 5.68 μg/mL (Figure 4). On the highest concentration of 1,600 μg/mL tested, it showed inhibition of 118.48 ± 4.26%. The value found higher than 100% may be explained through interactions between peanut oil and the analytical system. Sometimes the bioactive compounds in the oil reduce the base line absorbance. The resultant reflects an apparent inhibition greater than 100%. Similarly (20), reported inhibitory activities exceeding 100% for *α*-amylase and *α*-glucosidase in their study on methanolic and aqueous extracts of *Indigofera cordifolia* seeds and leaves at high extract concentrations. This phenomenon has been observed in similar studies, where complex interactions between the enzyme, substrate, and the oil’s bioactive components are thought to contribute to the observed values (41). The actual inhibitory effect of the oil remains significant, as indicated by the lower IC_50_ value when compared to acarbose, which showed an IC_50_ value of 3650.93 ± 10.70 μg/mL (Table 1). A low inhibitory activity of acarbose against *α*-amylase has also been observed in previous studies (21). This further confirms the strong inhibitory effect of peanut oil at much lower concentrations than the standard drug. The enhanced inhibition observed with peanut oil suggests that its bioactive compounds are highly effective in interacting with *α*-amylase. According to the docking results (Table 2; Figure 2), compounds such as stearic acid (−7.5729 kcal/mol) and oleic acid (−6.5865 kcal/mol) exhibit strong binding affinities through hydrogen bonds and hydrophobic interactions. These interactions likely contribute to the oil’s superior *α*-amylase inhibitory activity, indicating its potential as a natural anti-diabetic agent.

**Table 1:** Inhibitory potential of peanut oil and acarbose on α-amylase and α-glucosidase.

Compound	Concentration (μg/ml)	α-amylase inhibition (%)	Concentration (μg/ml)	α-glucosidase inhibition (%)
Peanut Oil	12.5	23.16 ± 1.99	15.62	1.34 ± 1.77
	50	27.39 ± 3.30	31.25	2.01 ± 3.56
	100	35.49 ± 3.49	62.5	6.60 ± 1.53
	200	47.76 ± 1.01	125	9.17 ± 1.11
	400	68.21 ± 2.15	250	14.13 ± 4.88
	800	93.27 ± 6.83	500	16.28 ± 2.51
	1,600	118.48 ± 4.26	1,000	25.86 ± 0.65
Acarbose	62.5	7.76 ± 0.17	12.5	8.36 ± 2.08
	125	8.08 ± 0.30	25	21.55 ± 7.96
	250	9.46 ± 0.11	50	34.65 ± 5.90
	500	10.70 ± 0.96	100	36.50 ± 4.02
	1,000	31.81 ± 2.89	200	39.46 ± 2.87
	2000	37.21 ± 3.54	400	48.64 ± 9.65
	4,000	53.05 ± 1.59	800	61.04 ± 4.29

**Table 2:** Tabular data of docking results of the top five active compounds from peanut oil against α-amylase (PDB ID: 2QV4), showing binding energies, hydrogen bond interactions, hydrophobic interactions, and electrostatic interactions within the enzyme’s active site.

Compound	Binding energy (kcal/mol)	Hydrogen bond interactions (Distance Å)	Hydrophobic interactions	Electrostatic interactions
Co-crystallized ligand (QV4)	−10.2266	Ala106 (2.96, 2.09, 2.79), Gly104 (2.67), Asn105 (2.16), Thr163 (1.97, 2.83), Gln63 (2.00, 1.91), Trp59 (1.82), Asp300 (2.96, 1.58), Arg195 (2.53), His299 (2.04), Glu233 (2.59, 2.21), Asp197 (1.59), His201 (1.80, 2.94), His305 (3.15)	-	Asp300, Glu233, Asp197
Acarbose	−8.6659	Glu233 (3.07, 2.92), His201 (2.95), Lys200 (2.16), Asp300 (2.61, 2.89, 2.38), Thr163 (1.97, 2.06), Gln63 (2.10)	-	-
Stearic Acid	−7.5729	Gly164 (2.58), Asn105 (2.30), Ala106 (2.42)	His201, Leu162, Tyr62, Leu165, Trp59	-
Docosanoic Acid	−7.1463	Lys200 (2.45)	Val107, Ile51, Leu165, Trp59, Tyr62, Leu162, His201, Ile235	Lys200, Tyr151
Oleic Acid	−6.5865	Lys200 (2.50)	Trp59, Leu165, Leu162, His101	Lys200
Palmitic Acid	−6.4778	Lys200 (2.53)	Trp59, Leu165, Tyr62, Leu162, Ile235, His201, Lys200	Lys200
E, E, Z-1,3,12-Nonadecatriene-5,14-diol	−6.4158	Asp197	Leu162, Leu165, Trp59, Ile51, Val107	-

#### 3.3.2 α-glucosidase inhibition potential

**Figure 5:**
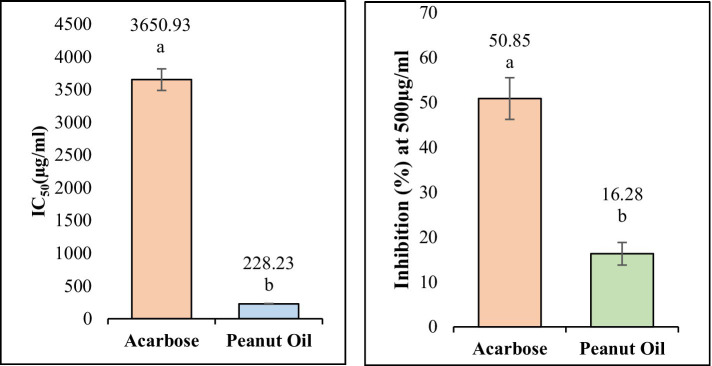
The IC_50_ values of peanut oil and acarbose for α-Amylase and α-glucosidase. Small letters inset (a, b) show a statistically significant difference (*p < 0.05*).

In contrast, peanut oil exhibited comparatively weaker inhibition of α-glucosidase. The IC_50_ value for peanut oil was found to be greater than 1,000 μg/mL, with a maximum inhibition of 25.86 ± 0.65% at the highest tested concentration (Table 1; Figure 5). This is markedly lower than the inhibition achieved by acarbose, which has an IC_50_ value of 405.77 ± 34.83 μg/mL and a maximum inhibition of 61.04% (Table 1) Previous studies (20) have also reported low inhibitory activity of seed extracts against *α*-glucosidase compared to the standard, acarbose. The reduced efficacy of peanut oil in inhibiting α-glucosidase may be attributed to less favorable interactions between its components and the enzyme. However, docking studies indicate that compounds included in this category, such as docosanoic acid (−6.3286 kcal/mol) and palmitic acid (−6.2753 kcal/mol), exhibit moderate binding energies and form less hydrogen bonds with the critical residues of *α*-glucosidase (Table 3; Figure 3). Based on the observations mentioned above, one may suggest that the peanut oil had an α-glucosidase inhibitory action; however, as compared to acarbose, the potential was very low due to its minimal inhibition rate against *α*-glucosidase. In the view of the observed activity, the present study is focused on *in vitro* and *in silico* analyses as a step necessary to understand its pharmacological potential.

**Table 3:** Tabular data of docking results of the top five active compounds from peanut oil against α-glucosidase (5NN8), showing binding energies, hydrogen bond interactions, hydrophobic interactions, and electrostatic interactions within the enzyme’s active site.

Compounds	Binding energy (kcal/mol)	Hydrogen bond interactions (Distance Å)	Hydrophobic interactions	Electrostatic interactions
Co-crystallized ligand (Acarbose)	−7.8732	Ala284 (2.11), Asp282 (1.71, 1.73), Arg600 (2.13), Asp616 (1.54, 2.59, 2.58, 2.74), Asp518 (2.30), His674 (2.10, 2.31), Asp404 (1.56, 2.87, 2.65, 1.66)	Trp481, Phe649, Trp376	Asp282, Asp616, Asp518
Oxiraneoctanoic acid, 3-octyl	−6.5120	His674 (2.12), Asp616 (2.70, 2.53)	Trp516, Phe525, Ala555	-
Docosanoic acid	−6.3286	His674 (2.03)	Trp618, Leu283, Ala284, Ala555, Trp481	Phe649
Palmitic acid	−6.2753	His674 (2.65)	Ala555, Trp376	Phe649
Eicosanoic acid	−6.2354	His674 (2.71)	Arg527, Ala555, Ala554, Met519, Trp418	Phe649
Tetracosanoic acid	5.8252	-	Val548, Ala555, Met519	Trp376, Trp481

To address the varying presentation of inhibition results, different concentration ranges were used for the α-amylase and α-glucosidase assays to accurately determine the IC₅₀ values for both the standard compound (acarbose) and peanut oil. The α-amylase assay showed a more potent inhibitory effect at lower concentrations, with the IC₅₀ value calculated at 228.23 ± 5.68 μg/mL, thus requiring smaller concentrations for meaningful inhibition values. In contrast, the α-glucosidase assay exhibited much lower inhibition at comparable concentrations of peanut oil, necessitating higher concentrations (e.g., 1,000 μg/mL) to observe significant inhibitory effects. This explains why inhibition percentages are used for the α-glucosidase assay, rather than IC₅₀ values, which would not have been accurate due to the less potent inhibition at lower concentrations. The use of percentages in the α-glucosidase assay ensures the presentation of meaningful data, reflecting the true inhibitory capacity of peanut oil at different concentrations. These differences in concentration ranges and result presentation are essential for accurately reflecting the varying inhibition profiles of peanut oil against these two enzymes. Similarly (22), also took the same approach. The α-amylase and α-glucosidase inhibitory activity of *Adiantum caudatum* and *Celosia argentea* extracts and fractions was investigated in the same manner, citing a wide concentration range in achieving IC₅₀ values for every enzyme because such extracts have differential potencies on enzymes under study.

## 4 Discussion

Peanuts and their derivatives, such as peanuts oil, have been increasingly recognized for their potential in managing hyperglycemia and diabetes (23). Peanuts oil is particularly noted for its high content of unsaturated fatty acids, which offer various health benefits, including inhibitory effects on key enzymes involved in glucose metabolism (24, 36). These unsaturated fatty acids act as competitive inhibitors, binding to specific sites on enzymes without affecting the enzyme’s maximum reaction rate (Vmax), thereby increasing the enzyme’s Km value (25). Because of their special mechanism, they can be used in conjunction with other diabetic therapies as a substitute for or addition to traditional oral hypoglycemic medications.

The present work reports a spectacular 51.85% extraction yield of peanut oil, in line with the findings of (26), who utilized a continuous phase-transition extraction procedure to attain a high yield while preserving oil quality. Likewise, Tu and Wu (27) illustrated the efficiency of these approaches by showing notable increases in oil yield using sophisticated extraction techniques. This alignment shows how crucial effective extraction methods are to bringing out the best in peanut oil’s therapeutic components.

The current investigation evaluated the inhibitory potential of El Oued peanut oil against *α*-amylase and α-glucosidase by combining *in vitro* and *in silico* methods. With an IC_50_ value of 228.23 ± 5.68 μg/mL, the *in vitro* data showed that peanut oil demonstrated a strong α-amylase inhibitory activity. The performance of this compound is much higher than that of acarbose, a standard inhibitor, which has an IC_50_ of 3650.93 ± 10.70 μg/mL (Table 1). The strong inhibitory potential of peanut oil may be attributed to its bioactive elements, such as oleic acid and stearic acid, which expressed strong binding interactions with *α*-amylase (Table 2; Figure 2). GC–MS analysis of the oil showed peaks corresponding to oleic acid, palmitic acid, and stearic acid, which have been extensively studied for their anti-diabetic potential, especially through the inhibition of key carbohydrate-hydrolyzing enzymes like *α*-amylase and α-glucosidase. These enzymes are responsible for the breakdown of complex carbohydrates into glucose, which can contribute to postprandial hyperglycemia. α-amylase inhibitors retard starch digestion in the small intestine; α-glucosidases inhibit the breakdown of disaccharides to monosaccharides and thus delay their absorption. Oleic acid, a prominent MUFA, has shown promise in improving insulin sensitivity and reducing blood glucose levels, potentially due to its impact on lipid metabolism and anti-inflammatory properties (28). Meanwhile, palmitic and stearic acids, though saturated, have shown moderate inhibition of these enzymes, helping to regulate glucose levels by delaying carbohydrate digestion and absorption. Together with minor compounds such as oxiraneoctanoic acid, these fatty acids may indicate that peanut oil can be a natural therapeutic agent in controlling type 2 diabetes by reducing postprandial blood glucose spikes. Gomes et al. (29) reported the potential effect of oleic acid in improving insulin sensitivity. Moreover, supplementation of *α*-linolenic acid improves insulin sensitivity in patients with type 2 diabetes (29). It has been reported also by Miyazawa et al. (30), that palmitic acid hydroxy stearic acids activate GPR40, which is involved in their beneficial effects on glucose homeostasis.

Using molecular docking tools, it was found that stearic acid showed a binding energy of −7.5729 kcal/mol and formed hydrogen bonds with residues like Gly164, Asn105, and Ala106, besides hydrophobic interactions with His201, Leu162, Tyr62, Leu165, and Trp59. This suggests a moderate inhibitory effect, consistent with previous studies where stearic and oleic acids showed similar α-amylase inhibition, albeit less potent than acarbose (31), leading to alteration of the enzyme’s active site, as a result inhibition of starch hydrolysis.

The enhanced inhibitory potential of these fatty acids might be achieved through their combination with other bioactive compounds, which can synergistically increase their binding affinity and overall inhibitory effect. Such research could provide promising strategies for hyperglycemia management, potentially leading to the development of new dietary supplements.

In contrast, peanut oil exhibited weaker inhibition of *α*-glucosidase compared to acarbose. With an IC_50_ value greater than 1,000 μg/mL and a maximum inhibition of 25.86 ± 0.65% (Table 1), peanut oil’s inhibitory activity was less potent than acarbose, which had an IC_50_ of 405.77 ± 34.83 μg/mL and achieved a maximum inhibition of 61.04%. The reduced effectiveness of peanut oil against *α*-glucosidase could be due to less favorable binding interactions with the enzyme. *In silico* analysis revealed that compounds such as oxiraneoctanoic acid, 3-octyl, exhibited a favorable binding energy of −6.5120 kcal/mol and formed hydrogen bonds with key residues His674 and Asp616, which are crucial for the enzyme’s catalytic activity (32). This interaction with Asp616 is particularly significant, as this residue participates in forming a glycosyl-enzyme intermediate during glucose hydrolysis.

Docosanoic acid and palmitic acid, binding energies of which were −6.3286 and −6.2753 kcal/mol respectively, also interacted with His674 but showed hydrophobic interactions with residues such as Trp618, Leu283, Phe649 among others. As indicated by such findings, these indirect interactions between the enzyme’s active site influence its activity upon changing the conformation of enzymes (33). However, tetracosanoic acid, although it had a binding energy of −5.8252 kcal/mol, did not make hydrogen bonds with key catalytic residues and mostly interacted via hydrophobic forces. Consequently, this poor interaction with essential residues led to less effective inhibition compared to the other compounds.

These results from this *in silico* docking study indicate that peanut oil compounds possess *α*-amylase and α-glucosidase inhibitory potentials to various degrees, which contribute to their anti-hyperglycemic activity. Fatty acids such as stearic acid, oleic acid, and palmitic acid showed moderate inhibition against α-amylase. This is supported by previous studies that have shown the modulatory role of these fatty acids in enzyme activity pertinent to carbohydrate metabolism. Stearic acid, for instance, has been shown to inhibit α-amylase by forming both hydrogen and hydrophobic interactions, which could slow the digestion of starch and reduce postprandial glucose spikes.

Similarly, compounds like oxiraneoctanoic acid and docosanoic acid displayed significant α-glucosidase inhibition, a key mechanism for delaying glucose absorption and managing hyperglycemia in type 2 diabetes. These compound bindings to the essential catalytic residues, such as Asp616 and His674, have similarities with previous studies that have shown the effectiveness of lipid-based inhibitors in reducing enzymatic activity (31). These findings therefore suggest that peanut oil, being rich in these bioactive compounds, might be of therapeutic benefit in the management of diabetes mellitus through natural means by exerting dual inhibition against *α*-amylase and α-glucosidase to control blood sugar levels.

The discrepancy between predictions *in silico* and results obtained through *in vitro* studies highlights these limitations of a computational model, unable to fully handle such biological complexities as the effects of solvents and molecular interactions that occur through a real context (32). While *in silico* models provide valuable theoretical insights, *in vitro* assays offer a more accurate assessment of biological efficacy, as the substantial differences between theoretical predictions and experimental data highlight the need for practical validation of enzyme inhibition studies. To bridge this gap, future research could explore the synergistic potential of peanut oil by combining it with other natural inhibitors to enhance its bioactivity, while *in vivo* models should also be employed to validate its therapeutic potential and facilitate the translation of these findings into clinical applications.

The present study provides an important baseline characterization of the anti-hyperglycemic potential of peanut oil phytochemicals through docking and *in vitro* analyses. Future studies should incorporate advanced computational approaches, such as binding free energy calculations (ΔG_bind), molecular dynamics simulations, *in silico* ADMET predictions, and DFT descriptors, to confirm and extend the present findings, providing deeper insights into the stability, pharmacological relevance, and therapeutic potential of these compounds.

## 5 Conclusion

*In vitro* assay of peanut oil extracted from the El Oued region of Algeria by Soxhlet method using n-hexane as a solvent showed high anti-hyperglycemic activity. Inhibitory activities of the oil against *α*-amylase and α-glucosidase were determined with appreciable inhibition of α-amylase. Its IC_50_ value (228.23 ± 5.68 μg/mL) was significantly low compared to that of the known α-amylase inhibitor, acarbose (IC_50_ = 3650.93 ± 10.70 μg/mL). GC–MS analysis revealed a total of 20 fatty acid compounds, contributing to 99.9% of the total composition of oil. These were grouped into three categories: saturated fatty acids (SFA), monounsaturated fatty acids (MUFA), and polyunsaturated fatty acids (PUFA). The identified bioactive compounds, such as stearic acid and oleic acid, likely contribute to the oil’s potent α-amylase inhibition through effective binding interactions. However, peanut oil exhibited a more modest inhibitory effect on α-glucosidase, with an IC_50_ value greater than 1,000 μg/mL and a maximum inhibition of 25.86 ± 0.65%. This level of inhibition is significantly lower compared to acarbose, which achieves an IC_50_ of 405.77 ± 34.83 μg/mL and a maximum inhibition of 61.04%. This suggests that peanut oil’s components interact less favorably with α-glucosidase, and compounds such as oxiraneoctanoic acid and palmitic acid, although they show some inhibitory activity, may not be as effective as acarbose in inhibiting α-glucosidase. Taken together, though peanut oil shows great potential as a natural α-amylase inhibitor and alternative to synthetic inhibitors, its inhibitory activity against α-glucosidase is rather weak. It involves implications in the sense that further studies are needed in order to explore the possible synergistic effects of the bioactive compounds of peanut oil on enhancing its overall anti-hyperglycemic activity. Further optimization of the composition of peanut oil itself or investigation on the combination of other agents to enhance its α-glucosidase inhibitory effects, so as to further prove its validity as a potential therapeutic agent in the management of hyperglycemia.

## Data Availability

The original contributions presented in the study are included in the article/Supplementary material, further inquiries can be directed to the corresponding author.

## References

[1] Sun H Saeedi P Karuranga S Pinkepank M Ogurtsova K Duncan BB. IDF diabetes atlas: global, regional and country-level diabetes prevalence estimates for 2021 and projections for 2045. Diabetes Res Clin Pract. (2022) 183:109119. doi: 10.1016/j.diabres.2021.109119, PMID: 34879977 PMC11057359

[2] Wang CCL Hess CN Hiatt WR Goldfine AB. Clinical update: cardiovascular disease in diabetes mellitus. Circulation. (2016) 133:2459–502. doi: 10.1161/circulationaha.116.022194 27297342 PMC4910510

[3] Davies MJ Aroda VR Collins BS Gabbay RA Green J Maruthur NM. Management of hyperglycemia in type 2 diabetes, 2022. A consensus report by the American Diabetes Association (ADA) and the European Association for the Study of diabetes (EASD). Diabetes Care. (2022) 45:2753–86. doi: 10.2337/dci22-0034, PMID: 36148880 PMC10008140

[4] Buse JB Wexler DJ Tsapas A Rossing P Mingrone G Mathieu C. 2019 update to: management of hyperglycemia in type 2 diabetes, 2018. A consensus report by the American Diabetes Association (ADA) and the European Association for the Study of diabetes (EASD). Diabetes Care. (2019) 43:487–93. doi: 10.2337/dci19-0066 31857443 PMC6971782

[5] Marmitt DJ Shahrajabian MH Goettert MI Rempel C. Clinical trials with plants in diabetes mellitus therapy: a systematic review. Expert Rev Clin Pharmacol. (2021) 14:735–47. doi: 10.1080/17512433.2021.1917380, PMID: 33884948

[6] Al-Bukhaiti WQ Al-Dalali S Noman A Qiu S Abed SM Qiu S-X. Response surface modeling and optimization of enzymolysis parameters for the in vitro antidiabetic activities of peanut protein hydrolysates prepared using two proteases. Foods. (2022) 11:3303. doi: 10.3390/foods11203303, PMID: 37431052 PMC9602261

[7] Sun X-M Ye H-Q Liu J-B Wu L Lin D-B Yu Y-L. Assessment of anti-diabetic activity of peanut shell polyphenol extracts. J Zhejiang Univ Sci B. (2018) 19:764–75. doi: 10.1631/jzus.b1700401, PMID: 30269444 PMC6194356

[8] Akter F Jahan N Sultana N. Effect of Peanut (*Arachis Hypogaea* L.) on fasting blood glucose and Hba1c in Alloxan induced diabetic male rats. J Bangladesh Soc Physiol. (2014) 9:48–53. doi: 10.3329/jbsp.v9i2.22796

[9] Proença C Ribeiro D Freitas M Fernandes E. Flavonoids as potential agents in the management of type 2 diabetes through the modulation of α-amylase and α-glucosidase activity: a review. Crit Rev Food Sci Nutr. (2021) 62:3137–207. doi: 10.1080/10408398.2020.1862755 33427491

[10] Hua F Zhou P Wu H-Y Chu G-X Xie Z-W Bao G-H. Inhibition of α-glucosidase and α-amylase by flavonoid glycosides from lu’an GuaPian tea: molecular docking and interaction mechanism. Food Funct. (2018) 9:4173–83. doi: 10.1039/c8fo00562a, PMID: 29989631

[11] Riyaphan J Pham D-C Leong MK Weng C-F. *In silico* approaches to identify polyphenol compounds as α-glucosidase and α-amylase inhibitors against type-II diabetes. Biomolecules. (2021) 11:1877. doi: 10.3390/biom11121877, PMID: 34944521 PMC8699780

[12] Ramkumar KM Thayumanavan B Palvannan T Rajaguru P. Inhibitory effect of *Gymnema montanum* leaves on α-glucosidase and α-amylase activity and their relationship with polyphenolic content. Med Chem Res. (2009) 19:948–61. doi: 10.1007/s00044-009-9241-5

[13] Djeghim H Bellil I Khelifi D. Genetic diversity of the Algerian peanut population analyzed using morphological markers and seed storage proteins. Proc Appl Bot Genet Breed. (2021) 182:111–24. doi: 10.30901/2227-8834-2021-3-111-124

[14] Djeghim H Bellil I Boudchicha RH Boumegoura A Khelifi D. First records on genetic diversity and population structure of Algerian peanut (*Arachis hypogaea*) using microsatellite markers. Plant Mol Biol Report. (2021) 40:136–47. doi: 10.1007/s11105-021-01305-7

[15] Badwaik LS Prasad K Deka SC. Optimization of extraction conditions by response surface methodology for preparing partially defatted peanut. Int Food Res J. (2012) 19:341–6.

[16] Mahfoud F Assaf JC Elias R Debs E Louka N. Defatting and defatted peanuts: a critical review on methods of oil extraction and consideration of solid matrix as a by-product or intended target. PRO. (2023) 11:2512. doi: 10.3390/pr11082512

[17] Bourgou S Rebey IB Ben Kaab S Hammami M Dakhlaoui S Sawsan S. Green solvent to substitute hexane for bioactive lipids extraction from black cumin and basil seeds. Foods. (2021) 10:1493. doi: 10.3390/foods10071493 34203148 PMC8308025

[18] Zengin G Sarikurkcu C Aktumsek A Ceylan R Ceylan O. A comprehensive study on phytochemical characterization of *Haplophyllum myrtifolium* Boiss. Endemic to Turkey and its inhibitory potential against key enzymes involved in Alzheimer, skin diseases, and type II diabetes. Ind Crop Prod. (2014) 53:244–51. doi: 10.1016/j.indcrop.2013.12.043

[19] Lordan S Smyth TJ Soler-Vila A Stanton C Ross RP. The α-amylase and α-glucosidase inhibitory effects of Irish seaweed extracts. Food Chem. (2013) 141:2170–6. doi: 10.1016/j.foodchem.2013.04.123, PMID: 23870944

[20] Khatri DK Juvekar AR. Α-Glucosidase and α-amylase inhibitory activity of *Indigofera cordifolia* seeds and leaves extract. Int J Pharm Pharm Sci. (2014) 6:152–5.

[21] Sandeli AEK Khiri-Meribout N Benzerka S Gürbüz N Dündar M Karcı H. Silver (I)-N-heterocyclic carbene complexes: synthesis and characterization, biological evaluation of anti-cholinesterase, anti-α-amylase, anti-lipase, and antibacterial activities, and molecular docking study. Inorg Chim Acta. (2021) 525:120486. doi: 10.1016/j.ica.2021.120486

[22] Telagari M Hullatti K. In vitro α-amylase and α-glucosidase inhibitory activity of *Adiantum caudatum* Linn. and *Celosia argentea* Linn. Extracts and fractions. Indian J Pharmacol. (2015) 47:425–9. doi: 10.4103/0253-7613.161270 26288477 PMC4527066

[23] Çiftçi S Suna G. Functional components of peanuts (*Arachis hypogaea* L.) and health benefits: a review. Future Foods. (2022) 5:100140. doi: 10.1016/j.fufo.2022.100140

[24] Farazi M Houghton MJ Murray M Williamson G. A systematic review of the inhibitory effect of extracts from edible parts of nuts on α-glucosidase activity. Food Funct. (2023) 14:5962–76. doi: 10.1039/d3fo00328k, PMID: 37306209

[25] Teng H Chen L. Α-Glucosidase and α-amylase inhibitors from seed oil: a review of liposoluble substances to treat diabetes. Crit Rev Food Sci Nutr. (2017) 57:3438–48. doi: 10.1080/10408398.2015.1129309 26854322

[26] Ma Y Lai C Xu C Zhang K Liu Y Cao Y. Novel low-temperature continuous phase-transition extraction process for efficient peanut oil production. J Food Meas Charact. (2024) 18:6721–35. doi: 10.1007/s11694-024-02685-6

[27] Tu J Wu W. An advanced pilot method of separating peanut oils with high quality based on aqueous extraction. Sep Sci Technol. (2020) 55:739–51. doi: 10.1080/01496395.2019.1569691

[28] Palomer X Pizarro-Delgado J Barroso E Vázquez-Carrera M. Palmitic and oleic acid: the yin and yang of fatty acids in type 2 diabetes mellitus. Trends Endocrinol Metab. (2018) 29:178–90. doi: 10.1016/j.tem.2017.11.009, PMID: 29290500

[29] Gomes PM Hollanda-Miranda WR Beraldo RA Castro AVB Geloneze B Foss MC. Supplementation of α-linolenic acid improves serum adiponectin levels and insulin sensitivity in patients with type 2 diabetes. Nutrition. (2015) 31:853–7. doi: 10.1016/j.nut.2014.12.028, PMID: 25933493

[30] Miyazawa M Yagi N Taguchi K. Inhibitory compounds of α-glucosidase activity from *Arctium lappa* L. J Oleo Sci. (2005) 54:589–94. doi: 10.5650/jos.54.589

[31] Ahmad P Alvi SS Iqbal J Khan MS. Identification and evaluation of natural organosulfur compounds as potential dual inhibitors of α-amylase and α-glucosidase activity: an *in-silico* and *in-vitro* approach. Med Chem Res. (2021) 30:2184–202. doi: 10.1007/s00044-021-02799-2

[32] Bulusu G Desiraju GR. Strong and weak hydrogen bonds in protein–ligand recognition. J Indian Inst Sci. (2019) 100:31–41. doi: 10.1007/s41745-019-00141-9

[33] Sanphui P Rajput L Gopi SP Desiraju GR. New multi-component solid forms of anti-cancer drug erlotinib: role of auxiliary interactions in determining a preferred conformation. Acta Crystallogr Sect B Struct Sci Cryst Eng Mater. (2016) 72:291–300. doi: 10.1107/s2052520616003607 27240760

[34] Adams RP. Identification of essential oil components by gas chromatography/mass spectroscopy. 4th ed. Carol Stream, IL: Allured Pub Corp (2007).

[35] Bayer FL Goodley PC Gordon M. Rapid gas chromatographic separation of diastereomeric dihalo-butanes, pentanes, and hexanes. J Chromatogr Sci. (1973) 11:443–6.

[36] Hu H Shi A Liu H Liu L Fauconnier ML Wang Q. Study on key aroma compounds and their precursors of peanut oil prepared with normal- and high-oleic peanuts. Foods. (2021) 10:3036. doi: 10.3390/foods10123036 34945587 PMC8701944

[37] Joseph B Jini D. Antidiabetic effects of *Momordica charantia* (bitter melon) and its medicinal potency. Asian Pac J Trop Dis. (2013) 3:93–102. doi: 10.1016/s2222-1808(13)60052-3

[38] Kim J Noh W Kim A Choi Y Kim Y-S. The effect of fenugreek in type 2 diabetes and prediabetes: a systematic review and meta-analysis of randomized controlled trials. Int J Mol Sci. (2023) 24:13999. doi: 10.3390/ijms241813999, PMID: 37762302 PMC10531284

[39] Mastelic J Jerkovic I Mesic M. Volatile constituents from flowers, leaves, bark and wood of *Prunus mahaleb* L. Flavour Fragr J. (2006) 21:306–13. doi: 10.1002/ffj.1596

[40] Neelakantan N Narayanan M De Souza RJ Van Dam RM. Effect of fenugreek (*Trigonella foenum-graecum* L.) intake on glycemia: a meta-analysis of clinical trials. Nutr J. (2014) 13:7. doi: 10.1186/1475-2891-13-7, PMID: 24438170 PMC3901758

[41] Yin Z Zhang W Feng F Zhang Y Kang W. Α-glucosidase inhibitors isolated from medicinal plants. Food Sci Human Wellness. (2014) 3:136–74. doi: 10.1016/j.fshw.2014.11.003

