# Supplementary material for: Article antidiabetic potential of peanut oil: inhibiting α-amylase and α-glucosidase using identified phytochemicals through in vitro and in silico studies
Source: Front Nutr. 2025 Oct 7;12:1592468. doi: 10.3389/fnut.2025.1592468 (PMC12537361; doi:10.3389/fnut.2025.1592468)
Supplement: Supplementary file 1 [file Table_1.docx]

Supplementary Material

Article Antidiabetic Potential of Peanut Oil: Inhibiting α-Amylase and α-Glucosidase Using Identified Phytochemicals Through *In Vitro* and *In Silico* Studies

Djamila Benouchenne^1,2^, Hanène Djeghim^1,3^, Ouided Benslama^4^, Huda Alsaeedi^5^, David Cornu^6^, Mikhael Bechelany^6^ and Ahmed Barhoum^7,8*^

^1^Laboratoire de Génétique, Biochimie et Biotechnologie végétale, Faculté des Sciences de la Nature et de la vie, Université des Frères Mentouri Constantine 1, 25000 Constantine, Algeria

^2^ Higher National School of Biotechnology Taoufik KHAZNADAR, nouveau Pôle universitaire Ali Mendjli, BP. E66, Constantine, 25100, Algeria. [dbenouchenne1992@gmail.com](mailto:dbenouchenne1992@gmail.com)

^3^Biochemistry Laboratory, Biotechnology Research Center (CRBt), P.O. Box E73, Nouvelle Ville Ali Mendjeli, Constantine 25016, Algeria. [livenanou@hotmail.com](mailto:livenanou@hotmail.com)

^4^Department of Natural and Life Sciences, Faculty of Exact Sciences and Natural and Life Sciences, Larbi Ben M’Hidi University, Oum El Bouaghi 04000, Algeria. [benslama.wided@hotmail.fr](mailto:benslama.wided@hotmail.fr)

^5^Department of Chemistry, College of Science, King Saud University, Riyadh, Saudi Arabia. [halsaeedi@ksu.edu.sa](mailto:halsaeedi@ksu.edu.sa)

^6^Institut Européen des Membranes, IEM, UMR-5635, University Montpellier, ENSCM, CNRS, Place Eugene Bataillon, Montpellier, France. [david.cornu@umontpellier.fr](mailto:david.cornu@umontpellier.fr), [mikhael.bechelany@umontpellier.fr](mailto:mikhael.bechelany@umontpellier.fr)

^7^NanoStruc Research Group, Chemistry Department, Faculty of Science, Helwan University, Cairo, Egypt.

^8^ School of Chemical and BioPharmaceutical Sciences, Technological University Dublin, Grangegorman Campus, 7, Dublin, D07 ADY7, Ireland

*** Correspondence:**Ahmed Barhoum
[ahmed.barhoum@science.helwan.edu.eg](mailto:ahmed.barhoum@science.helwan.edu.eg), [ahmed.barhoum@tudublin.ie](mailto:ahmed.barhoum@tudublin.ie)

**Table S1.** Peak Identification and Quantification of Compounds in Peanut Oil (*Arachis hypogaea* L.) by Gas Chromatography–Mass Spectrometry (GC-MS) Based on Wiley mass spectra Matching.

| **PK** | **Compound** | **RT (min)** | **Characteristic mass (m/z)** | **% Max** | | **Conc (% of total)** | **Category** | **References** |  |
| --- | --- | --- | --- | --- | --- | --- | --- | --- | --- |
| 1 | n-Caproic acid | 6.51 | 59.0, 74.0, 87.0, 99.0 | 0.26 | | 0.11 | SFA | Nicolescu, 2017 ; Bong et al., 2018 |  |
| 2 | Heptanoic acid | 8.85 | 55.0, 74.0, 87.0, 101.0, 113.0 | 0.23 | | 0.09 | SFA | Bong et al., 2018 |  |
| 3 | n-Caprylic acid | 10.89 | 55.0, 74.0, 87.0, 101.0, 115.0, 127.0, 143.0, 158.0 | 1.56 | | 0.65 | SFA | Bong et al., 2018 |  |
| 4 | Azela aldehydic acid | 18.79 | 55.0, 74.0, 87.0, 111.0, 129.0, 143.0, 158.1 | 0.64 | | 0.27 | SFA | Bong et al., 2018 |  |
| 5 | Nonanedioic acid | 22.49 | 55.0, 74.0, 96.0, 111.0, 128.0, 152.0, 185.1 | 2.14 | | 0.89 | SFA | Villette et al., 2018 ; Shokrollahi et al., 2022 |  |
| 6 | Palmitic acid | 35.21 | 55.0, 74.0, 97.0, 115.0, 143.1, 167.1, 185.1, 209.1, 227.2, 270.2 | 64.08 | | 26.90 | SFA | Gołębiowski et al., 2013 ; Madhu & Sharma, 2017 ; Bong et al., 2018 |  |
| 7 | α-linolenic acid | 40.30 | 67.0, 95.0, 123.0, 150.0, 178.1, 220.1, 263.2, 294.2 | 5.66 | | 2.37 | PUFA | Yang et al., 2014 |  |
| 8 | Oleic acid | 40.68 | 55.0, 74.0, 97.0, 123.0, 152.1, 180.1, 199.1, 222.2, 241.2, 264.2, 296.2 | 100.00 | | 41.98 | MUFA | Madhu & Sharma, 2017; Carrillo et al., 2018 ; Waheed et al., 2019 |  |
| 9 | Stearic acid | 41.32 | 53.0, 74.0, 97.0, 121.0, 143.0, 171.0, 199.1, 227.1, 255.2, 298.2 | 19.34 | | 8.11 | SFA | Madhu & Sharma, 2017; Carrillo et al., 2018 |  |
| 10 | 4-Methyl-exo-tricyclo [6.2.1.0(2.7)] undecane | 45.16 | 55.0, 68.0, 81.0, 95.0, 108.0, 121.0, 136.0, 149.0, 164.0, 178.1, 199.0 | 1.41 | | 0.59 | Others | Swithenbank & Whiting, 1963; US Patent US5538944A, 1995 |  |
| 11 | 6-Methyl Octahydro Coumarin | 45.46 | 55.0, 68.0, 81.0, 95.0, 109.0, 125.0, 139.0, 155.0, 168.0, 185.0, 200.0 | 1.32 | | 0.55 | Others | Lopez-Avila, 2011 |  |
| 12 | Iso-Iridomyrmecin | 45.73 | 55.0, 81.0, 95.0, 121.0, 135.0, 149.0, 167.0, 181.1, 199.1, 213.0 | 1.68 | | 0.70 | Others | NIST Chemistry WebBook, 2024a |  |
| 13 | Oxiraneoctanoic acid, 3-octyl | 46.03 | 55.0, 83.0, 109.0, 130.0, 155.0, 199.1, 245.2, 281.2 | 8.33 | | 3.49 | SFA | Hussein et al., 2016 |  |
| 14 | Oxiraneoctanoic acid, 3-octyl | 46.50 | 55.0, 87.0, 109.0, 130.1, 155.0, 199.1, 237.1, 263.2 | 6.32 | | 2.65 | MUFA | Hussein et al., 2016 |  |
| 15 | E, E, Z-1,3,12-Nonadecatriene-5,14-diol | 46.80 | 55.0, 83.0, 121.0, 144.0, 167.0, 199.1, 228.1, 250.2, 292.2, 324.2 | 6.37 | | 2.67 | SFA | Hadi et al., 2016 |  |
| 16 | Eicosanoic acid | 47.73 | 74.0, 97.0, 143.0, 171.0, 199.1, 227.1, 255.2, 283.2, 326.3 | 5.50 | | 2.30 | MUFA | Abubaker et al., 2021 |  |
| 17 | (2R)-2-(1,3-Dithian-2-yl) isoborneol | 48.49 | 55.0, 79.0, 97.0, 114.9, 133.0, 153.1, 183.1, 211.1, 251.2, 279.1 | 2.58 | | 1.08 | Others | NIST Chemistry WebBook, 2024b |  |
| 18 | Carbamic acid | 49.59 | 55.0, 83.0, 107.0, 127.0, 153.0, 185.1, 229.1, 279.1, 311.2 | 1.42 | | 0.59 | Others | Kadhim et al., 2016 |  |
| 19 | Docosanoic acid | 51.85 | 74.0, 97.0, 121.0, 143.0, 171.1, 199.1, 227.1, 255.2, 283.2, 311.2, 354.3 | 5.80 | | 2.43 | SFA | Waheed et al., 2019 ; Abubaker et al., 2021 |  |
| 20 | Tetracosanoic acid | 54.45 | 74.0, 101.0, 143.0, 171.0, 199.1, 241.1, 283.2, 311.2, 339.3, 382.3 | 2.24 | | 0.94 | SFA | Zayed et al., 2016 |  |
| **Category** | | | | | **Total (%)** | | | | |
| Saturated Fatty acids (SFA) | | | | | 47.18 | | | | |
| Unsaturated Fatty Acids (UFA) | | | | | 49.31 | | | | |
| Monounsaturated Fatty Acids (MUFA) | | | | | 46.94 | | | | |
| Polyunsaturated Fatty acids (PUFA) | | | | | 2.38 | | | | |
| Others | | | | | 3.90 | | | | |
| **Total (%)** | | | | | 99.9 | | | | |

**Table S2.** Docking results of the co-crystallized ligand (QV4), acarbose, and compounds of peanut oil with alpha amylase (2QV4)

| **Compounds** | **Binding energy (Kcal/mol)** | **Hydrogen interactions**  **(Distance Å)** | **Hydrophobic interactions** | **Electrostatic interactions** |
| --- | --- | --- | --- | --- |
| Co-crystallized ligand (QV4) | -10.2266 | Ala106 (2.96, 2.09, 2.79), Gly104 (2.67), Asn105 (2.16), Thr163 (1.97, 2.83), Gln63 (2.00, 1.91), Trp59 (1.82), Asp300 (2.96, 1.58), Arg195 (2.53), His299 (2.04), Glu233 (2.59, 2.21), Asp197 (1.59), His201 (1.80, 2.94), His305 (3.15) | - | Asp300, Glu233, Asp197 |
| Acarbose | -8.6659 | Glu233 (3.07, 2.92), His201 (2.95), Lys200 (2.16), Asp300 (2.61, 2.89, 2.38), Thr163 (1.97, 2.06), Gln63 (2.10) | - | - |
| Stearic Acid | -7.5729 | Gly164 (2.58), Asn105 (2.30), Ala106 (2.42) | His201, Leu162, Tyr62, Leu165, Trp59 | - |
| Docosanoic Acid | -7.1463 | Lys200 (2.45) | Val107, Ile51, Leu165, Trp59, Tyr62, Leu162, His201, Ile235 | Lys200, Tyr151 |
| Oleic Acid | -6.5865 | Lys200 (2.50) | Trp59, Leu165, Leu162, His101 | Lys200 |
| Palmitic Acid | -6.4778 | Lys200 (2.53) | Trp59, Leu165, Tyr62, Leu162, Ile235, His201, Lys200 | Lys200 |
| E,E,Z-1,3,12-Nonadecatriene-5,14-diol | -6.4158 | Asp197 | Leu162, Leu165, Trp59, Ile51, Val107 | - |
| Oxiraneoctanoic acid, 3-octyl (SFA) | -6.3745 | Asp300(3.41) | Ile235, Trp59 | - |
| α-linolenic acid | -6.3425 | - | Leu165, His305, His101, Leu162, Trp59, Trp58 | Lys200 |
| Nonanedioic acid | -6.3122 | - | Leu62, Ala198 | Lys200, His101 |
| Eicosanoic acid | -6.2632 | - | His101, Leu162, Trp59, Leu165, Ala106 | Lys200 |
| Tetracosanoic acid | -6.1256 | - | Val107, Ile51, Trp59, Leu165, Leu162 | Lys200 |
| 6-Methyl Octahydro Coumarin | -4.8936 | Arg195 (2.81), His299 (1.97) | His305 | - |
| Oxiraneoctanoic acid | -4.8614 | His299 (1.88), Arg195(2.78) | His101, Leu165, Leu162 | Tyr62 |
| (2R)-2-(1,3-Dithian-2-yl)isoborneol | -4.8362 | Gln63 (2.37) | LEU165, His101, His305, Trp59 | - |
| n-Caprylic acid | -4.7778 | His299 (2.60), His299 (1.93) | Trp59, Leu165 | Tyr62, Arg195 |
| Azelaaldehydic acid | -4.7162 | Gnl63 (2.13) | His305 |  |
| 4-Methyl-exo-tricyclo[6.2.1.0(2.7)]undecane | -4.6614 | - | Leu165, Trp59, His305 | - |
| iso-Iridomyrmecin | -4.4453 | - | Tyr62, trp59, Trp58, Leu165, His101 | - |
| Heptanoic acid | -4.2112 | Gln63 (2.05) | Trp58, His305,Trp59 | - |
| n-Caproic acid | -4.0663 | Gln (2.50), Gln (2.50) | Trp58, His305, Trp59 | - |
| Carbamic acid | -2.7853 | - | - | His101 |

**Table S3.** Docking results of the co-crystallized ligand and compounds of peanut oil with alpha glucosidase (5NN8)

| **Compounds** | **Binding energy (Kcal/mol)** | **Hydrogen interactions**  **(Distance Å)** | **Hydrophobic interactions** | **Electrostatic interactions** |
| --- | --- | --- | --- | --- |
| Co-crystallized ligand (Acarbose) | -7.8732 | Ala284 (2.11), Asp282 (1.71, 1.73), Arg600 (2.13), Asp616 (1.54, 2.59, 2.58, 2.74), Asp518 (2.30), His674 (2.10, 2.31), Asp404 (1.56, 2.87, 2.65, 1.66) | Trp481, Phe649, Trp376 | Asp282, Asp616, Asp518 |
| Oxiraneoctanoic acid, 3-octyl | -6.5120 | His674 (2.12), Asp616 (2.70, 2.53) | Trp516, Phe525, Ala555 | - |
| Docosanoic acid | -6.3286 | His674 (2.03) | Trp618, Leu283, Ala284, Ala555, Trp481 | Phe649 |
| Palmitic acid | -6.2753 | His674 (2.65) | Ala555, Trp376 | Phe649 |
| Eicosanoic acid | -6.2354 | His674 (2.71) | Arg527, Ala555, Ala554, Met519, Trp418 | Phe649 |
| Tetracosanoic acid | 5.8252 | - | Val548, Ala555, Met519 | Trp376, Trp481 |
| Nonanedioic acid (SFA) | -6.1645 | - | Leu405, Ile441, Trp481, Phe649, Trp376, Ala555, Arg527 | - |
| E,E,Z-1,3,12-Nonadecatriene-5,14-diol (SFA) | -6.0241 | - | Ile441, Leu405, Trp481, Phe649, Trp376, Ala555, Arg527 | - |
| Stearic acid (SFA) | -5.8871 | Trp481 (2.51) | Trp481, Leu283 | - |
| α-linolenic acid  (PUFA) | -5.6826 | - | Ala284, Met519, Trp481, Leu405, Trp376 | - |
| Oleic acid (MUFA) | -5.4933 | His674 (2.33) | Met519, Ala555 | - |
| Azelaaldehydic acid (SFA) | -4.9040 | His674 (2.50), Asp616 (2.65) | Trp376, Phe649 | - |
| (2R)-2-(1,3-Dithian-2-yl)isoborneol (NFA) | -4.8537 | Asp616 (2.73), Gly615 (2.81), Arg600 (2.36) | His674, Phe649, Trp613, Trp516, Trp376, Trp481 | - |
| Oxiraneoctanoic acid (MUFA) | -4.6900 | Leu677 (2.90), Asp616 (2.58), Asp518 (2.90) | Phe649, Trp376, Trp481 | - |
| 6-Methyl Octahydro Coumarin ((NFA) | -4.7163 | His674 (1.91) | Trp481, Met519, Trp376 | - |
| n-Caprylic acid (SFA) | -4.3600 | His674 (2.36) | Met519, Trp481, Trp376 | - |
| 4-Methyl-exo-tricyclo[6.2.1.0(2.7)]undecane (SFA) | -4.3365 | - | Met519, Trp481, Ile441, Trp376, Phe649 | - |
| Heptanoic acid (SFA) | -4.2117 | His674 (2.09) | Trp481 | - |
| n-Caproic acid (SFA) | -4.1582 | His674 (2.61) | Trp481, Leu405, Trp375 | - |
| iso-Iridomyrmecin (NFA) | -4.0860 | Leu283 (1.94), Asp282 (2.87), Leu283 (2.79) | Ala555 | - |
| Carbamic acid (NFA) | -3.0661 | His674 (2.02), Phe649 (2.46), Asp616 (2.20) | - | - |

**Figure S2.** Mass Spectra of Peanut Oil Compounds Identified by GC-MS and Wiley Database Matching.

| **Comp** | **Structure** | **Compound mass spectra** | **Library mass spectra (Wiley)** | **Wiley Ref** | **Wiley CAS** | **Qual** |
| --- | --- | --- | --- | --- | --- | --- |
| 1 | n-Caproic acid |  |  | 26364 | 000106-70-7 | 90 |
| 2 | Heptanoic acid |  |  | 40220 | 000106-73-0 | 90 |
| 3 | n-Caprylic acid |  |  | 57744 | 000111-11-5 | 97 |
| 4 | Azela aldehydic acid |  |  | 95128 | 001931-63-1 | 91 |
| 5 | Nonanedioic acid |  |  | 138131 | 001732-10-1 | 94 |
| 6 | Palmitic acid |  |  | 213911 | 000112-39-0 | 99 |
| 7 | α-linolenic acid |  |  | 243126 | 017309-05-6 | 99 |
| 8 | Oleic acid |  |  | 245466 | 000112-62-9 | 99 |
| 9 | Stearic acid |  |  | 247763 | 000112-61-8 | 99 |
| 10 | 4-Methyl-exo-tricyclo [6.2.1.0(2.7)] undecane |  |  | 65539 | 000000-00-0 | 64 |
| 11 | 6-Methyl Octahydro Coumarin |  |  | 70735 | 000000-00-0 | 90 |
| 12 | Iso-Iridomyrmecin |  |  | 70602 | 000000-00-0 | 86 |
| 13 | Oxiraneoctanoic acid, 3-octyl |  |  | 263154 | 002566-91-8 | 93 |
| 14 | Oxiraneoctanoic acid, 3-octyl |  |  | 263158 | 006084-76-0 | 91 |
| 15 | E, E, Z-1,3,12-Nonadecatriene-5,14-diol |  |  | 243168 | 000000-00-0 | 30 |
| 16 | Eicosanoic acid |  |  | 277460 | 001120-28-1 | 99 |
| 17 | (2R)-2-(1,3-Dithian-2-yl) isoborneol |  |  | 215647 | 063865-77-0 | 35 |
| 18 | Carbamic acid |  |  | 93707 | 000940-36-3 | 35 |
| 19 | Docosanoic acid |  |  | 302033 | 000929-77-1 | 99 |
| 20 | Tetracosanoic acid |  |  | 321681 | 002442-49-1 | 99 |

**References**

1. **Waheed, A.; Hamid, F.S.; Madiha, B.; Seemab, A.; Naveed, A.; Nadia, K.; Sohail, A.; Saqib, M.; Hina, G.** (2019). GC-MS analysis of chemical components seed oil of Raphanus sativus L. MOJ Toxicol. 5(3), 112–118. <https://doi.org/10.15406/mojt.2019.05.00164>.
2. Nicolescu, T.O. (2017). Interpretation of mass spectra. *Mass Spectrom.* 6, 111–133. <https://doi.org/10.5702/massspectrometry.MS2017.111>.
3. Madhu, D., Sharma, Y.C. (2017). Synthesis of a reusable novel catalyst (β-tricalcium phosphate) for biodiesel production from a common Indian tribal feedstock. *Resour.-Eff. Technol.* 3, 144–157. <https://doi.org/10.1016/j.reffit.2017.02.004>.
4. Gołębiowski, M., Czerwonka, R., Florek, M., Herczek, A., Stepnowski, P. (2013). The antifungal activity of the cuticular and internal fatty acid methyl esters and alcohols in *Calliphora vomitoria*. *Parasitol.* 140, 972–985. <https://doi.org/10.1017/S003118201300012X>.
5. Bong, A.M.; Ndikontar, M.K.; Ndifon, P.T.; Sani, Y.M. (2018). Synthesis and characterisation of biodiesel from Cameroon palm kernel seed oil. *Asian J. Biotechnol. Bioresour. Technol.* 3, 1–17. <https://doi.org/10.9734/AJB2T/2018/40200>.
6. Carrillo, W.; Greffa, J.; Vinueza, D.; Álvarez, M.; Silva, M.; Carpio, C.; Morales, D. (2018). Fatty acids content of kahai (Caryodendron orinocense Karst) seeds cultivated in Amazonian of Ecuador. Asian J. Pharm. Clin. Res. 11, 399–402. <https://doi.org/10.22159/ajpcr.2018.v11i2.16109>.
7. Yang, Q.; Cao, W.; Zhou, X.; Cao, W.; Xie, Y.; Wang, S. (2014). Anti-thrombotic effects of α-linolenic acid isolated from *Zanthoxylum bungeanum* Maxim seeds. *BMC Complement. Altern. Med.* 14, 348. <https://doi.org/10.1186/1472-6882-14-348>.
8. Shokrollahi, N.; Ho, C.L.; Mohd Zainudin, N.A.I.; Abdul Wahab, M.A.B.; Wong, M.Y. (2022). Plant defense inducers and antioxidant metabolites produced during oil palm-Ganoderma boninense interaction in vitro. Chemistry Africa 6, 499–511. <https://doi.org/10.1007/s42250-022-00501-6>.
9. Villette, C.; Zumsteg, J.; Schaller, H.; Heintz, D. (2018). Non-targeted metabolic profiling of BW312 Hordeum vulgare semi-dwarf mutant using UHPLC coupled to QTOF high resolution mass spectrometry. Sci. Rep. 8, 13178. <https://doi.org/10.1038/s41598-018-31593-1>.
10. Kadhim, M.J.; Mohammed, G.J.; Hussein, H.M. (2016). Analysis of bioactive metabolites from Candida albicans using GC-MS and evaluation of antibacterial activity. Int. J. Pharm. Clin. Res. 8, 655–670.
11. Zayed, M.A.; Abd El-Kareem, M.S.M.; Zaky, N.H.S. (2016). Gas chromatography–mass spectrometry studies of waste vegetable mixed and pure used oils and its biodiesel products. J. Pharm. Appl. Chem. 2, 30–37. <https://doi.org/10.18576/jpac/030204>.
12. Abubaker, M.A.; Mohammed, A.A.A.; Farah, A.A.M.; Zhang, J. (2021). Phytochemical screening by using GC-MS and FTIR spectrum analysis of fixed oil from Sudanese Ziziphus spina-christi seeds. Eurasian Chem. Commun. 3, 244–256. <https://doi.org/10.22034/ecc.2021.273055.1137>.
13. Hadi, M.Y.; Mohammed, G.J.; Hameed, I.H. (2016). Analysis of bioactive chemical compounds of Nigella sativa using gas chromatography–mass spectrometry. J. Pharmacogn. Phytother. 8, 8–24. <https://doi.org/10.5897/JPP2015.0364>.
14. Hussein, H.J.; Hadi, M.Y.; Hameed, I.H. (2016). Study of chemical composition of Foeniculum vulgare using Fourier transform infrared spectrophotometer and gas chromatography–mass spectrometry. J. Pharmacogn. Phytother. 8, 60–89. <https://doi.org/10.5897/JPP2015.0372>.
15. Lopez-Avila, V. (2011). Mass Spectral Fragmentation Studies of Coumarin-Type Compounds Using GC High-Resolution MS. Open Analytical Chemistry Journal, 5, 1–9.
16. Swithenbank, C & Whiting, M. C. (1963). Some derivatives of tricyclo[6.2.1.0²,⁷]undecane; the thermolysis of exo,exo-3-diazotricyclo[6.2.1.0²,⁷]undecane. Journal of the Chemical Society (Resumed), 4573–4578. <https://doi.org/10.1039/JR9630004573>.
17. US Patent US5538944A (1995). Composition containing a preponderant amount of (1RS,5RS)-5-methyl-exo-tricyclo[6.2.1.0²,⁷]undecan-4-one and its use in perfumery. <https://patents.google.com/patent/US5538944A/en>.
18. NIST Chemistry WebBook. (2024a). Iso-Iridomyrmecin mass spectrum. National Institute of Standards and Technology. <https://webbook.nist.gov/cgi/cbook.cgi?ID=C485438&Mask=200>.
19. NIST Chemistry WebBook. (2024b). (2R)-2-(1,3-Dithian-2-yl) isoborneol mass spectrum. National Institute of Standards and Technology. <https://pubchem.ncbi.nlm.nih.gov/compound/101500>.
